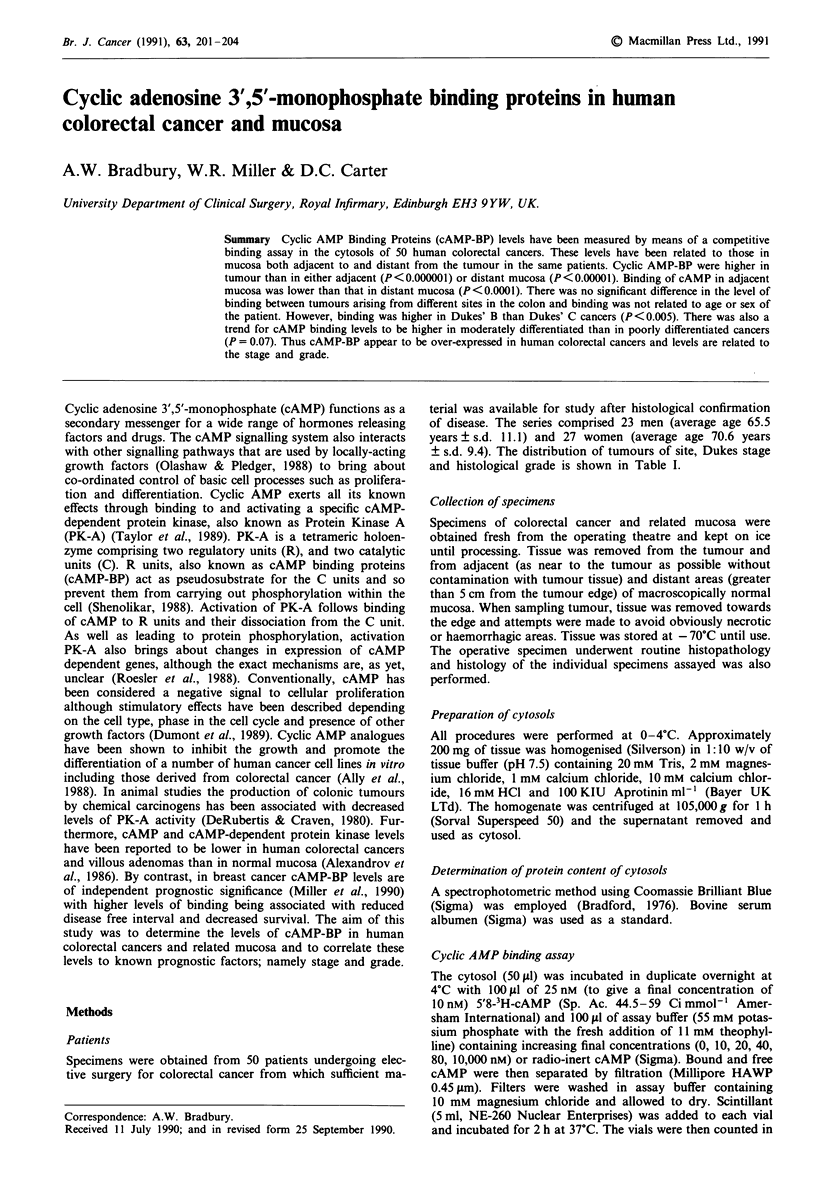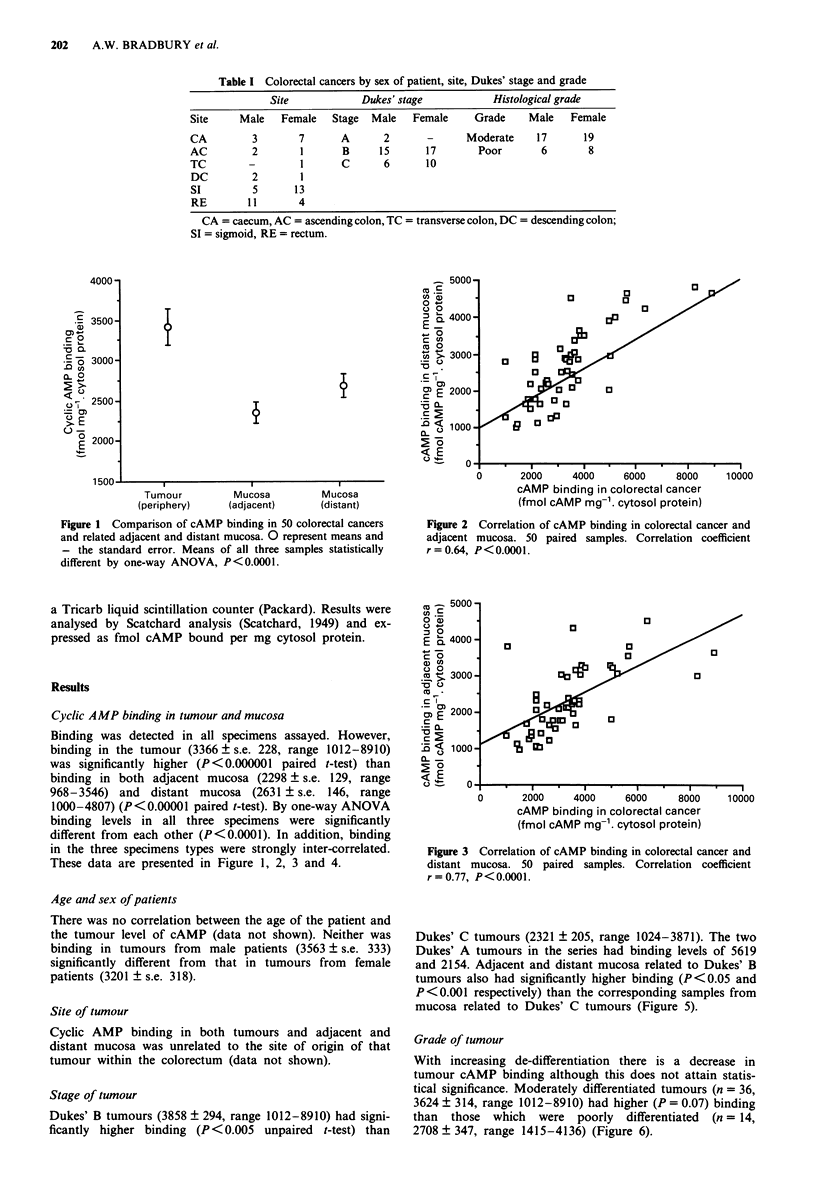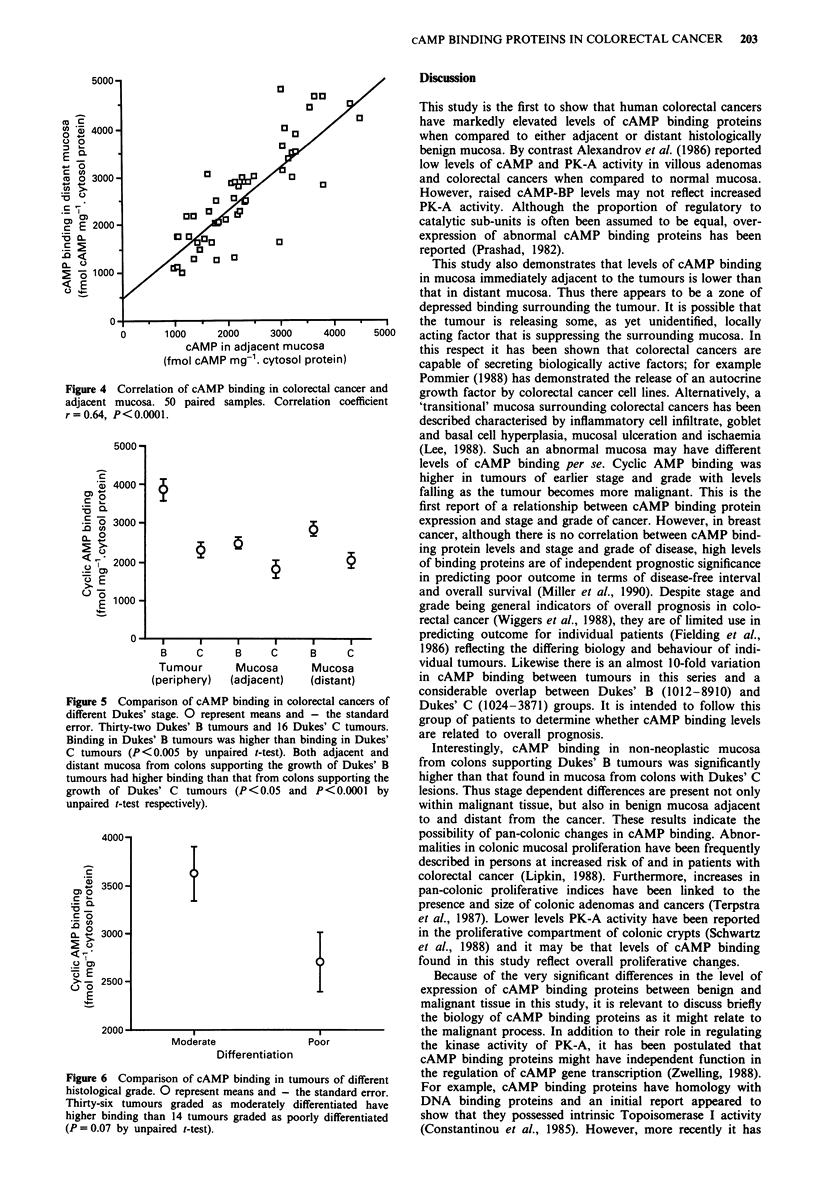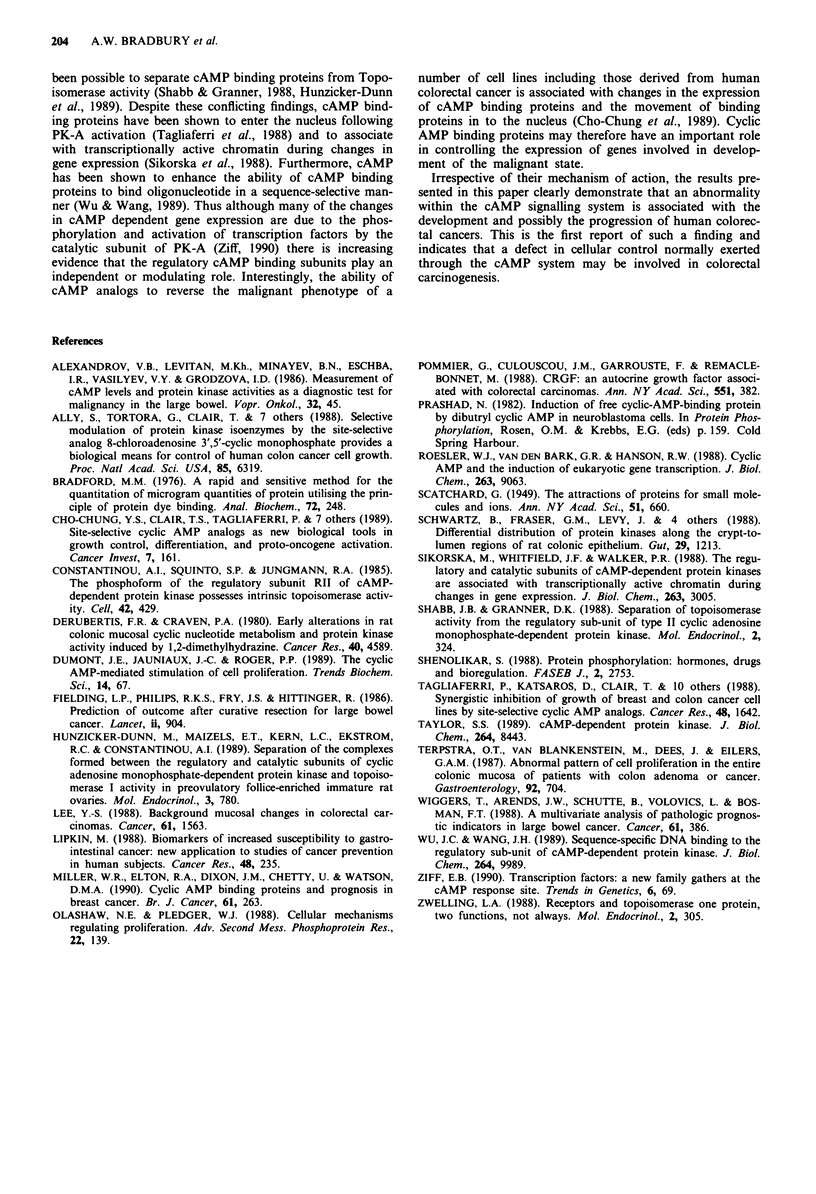# Cyclic adenosine 3',5'-monophosphate binding proteins in human colorectal cancer and mucosa.

**DOI:** 10.1038/bjc.1991.49

**Published:** 1991-02

**Authors:** A. W. Bradbury, W. R. Miller, D. C. Carter

**Affiliations:** University Department of Clinical Surgery, Royal Infirmary, Edinburgh, UK.

## Abstract

Cyclic AMP Binding Proteins (cAMP-BP) levels have been measured by means of a competitive binding assay in the cytosols of 50 human colorectal cancers. These levels have been related to those in mucosa both adjacent to and distant from the tumour in the same patients. Cyclic AMP-BP were higher in tumour than in either adjacent (P less than 0.000001) or distant mucosa (P less than 0.00001). Binding of cAMP in adjacent mucosa was lower than that in distant mucosa (P less than 0.0001). There was no significant difference in the level of binding between tumours arising from different sites in the colon and binding was not related to age or sex of the patient. However, binding was higher in Dukes' B than Dukes' C cancers (P less than 0.005). There was also a trend for cAMP binding levels to be higher in moderately differentiated than in poorly differentiated cancers (P = 0.07). Thus cAMP-BP appear to be over-expressed in human colorectal cancers and levels are related to the stage and grade.


Br. J. Cancer (1991), 63, 201-204 © Macmillan Press Ltd., 1991

### Cyclic adenosine 3′,5′-monophosphate binding proteins in human colorectal cancer and mucosa

A.W. Bradbury, W.R. Miller & D.C. Carter

*University Department of Clinical Surgery, Royal Infirmary, Edinburgh EH3 9YW, UK.*

**Summary** Cyclic AMP Binding Proteins (cAMP-BP) levels have been measured by means of a competitive binding assay in the cytosols of 50 human colorectal cancers. These levels have been related to those in mucosa both adjacent to and distant from the tumour in the same patients. Cyclic AMP-BP were higher in tumour than in either adjacent ($P < 0.000001$) or distant mucosa ($P < 0.00001$). Binding of cAMP in adjacent mucosa was lower than that in distant mucosa ($P < 0.0001$). There was no significant difference in the level of binding between tumours arising from different sites in the colon and binding was not related to age or sex of the patient. However, binding was higher in Dukes' B than Dukes' C cancers ($P < 0.005$). There was also a trend for cAMP binding levels to be higher in moderately differentiated than in poorly differentiated cancers ($P = 0.07$). Thus cAMP-BP appear to be over-expressed in human colorectal cancers and levels are related to the stage and grade.

Cyclic adenosine 3′,5′-monophosphate (cAMP) functions as a secondary messenger for a wide range of hormones releasing factors and drugs. The cAMP signalling system also interacts with other signalling pathways that are used by locally-acting growth factors (Olashaw & Pledger, 1988) to bring about co-ordinated control of basic cell processes such as proliferation and differentiation. Cyclic AMP exerts all its known effects through binding to and activating a specific cAMP-dependent protein kinase, also known as Protein Kinase A (PK-A) (Taylor *et al.*, 1989). PK-A is a tetrameric holoenzyme comprising two regulatory units (R), and two catalytic units (C). R units, also known as cAMP binding proteins (cAMP-BP) act as pseudosubstrate for the C units and so prevent them from carrying out phosphorylation within the cell (Shenolikar, 1988). Activation of PK-A follows binding of cAMP to R units and their dissociation from the C unit. As well as leading to protein phosphorylation, activation PK-A also brings about changes in expression of cAMP dependent genes, although the exact mechanisms are, as yet, unclear (Roesler *et al.*, 1988). Conventionally, cAMP has been considered a negative signal to cellular proliferation although stimulatory effects have been described depending on the cell type, phase in the cell cycle and presence of other growth factors (Dumont *et al.*, 1989). Cyclic AMP analogues have been shown to inhibit the growth and promote the differentiation of a number of human cancer cell lines *in vitro* including those derived from colorectal cancer (Ally *et al.*, 1988). In animal studies the production of colonic tumours by chemical carcinogens has been associated with decreased levels of PK-A activity (DeRubertis & Craven, 1980). Furthermore, cAMP and cAMP-dependent protein kinase levels have been reported to be lower in human colorectal cancers and villous adenomas than in normal mucosa (Alexandrov *et al.*, 1986). By contrast, in breast cancer cAMP-BP levels are of independent prognostic significance (Miller *et al.*, 1990) with higher levels of binding being associated with reduced disease free interval and decreased survival. The aim of this study was to determine the levels of cAMP-BP in human colorectal cancers and related mucosa and to correlate these levels to known prognostic factors; namely stage and grade.

### Methods

### *Patients*

Specimens were obtained from 50 patients undergoing elective surgery for colorectal cancer from which sufficient material was available for study after histological confirmation of disease. The series comprised 23 men (average age 65.5 years ± s.d. 11.1) and 27 women (average age 70.6 years ± s.d. 9.4). The distribution of tumours of site, Dukes stage and histological grade is shown in Table I.

### *Collection of specimens*

Specimens of colorectal cancer and related mucosa were obtained fresh from the operating theatre and kept on ice until processing. Tissue was removed from the tumour and from adjacent (as near to the tumour as possible without contamination with tumour tissue) and distant areas (greater than 5 cm from the tumour edge) of macroscopically normal mucosa. When sampling tumour, tissue was removed towards the edge and attempts were made to avoid obviously necrotic or haemorrhagic areas. Tissue was stored at −70°C until use. The operative specimen underwent routine histopathology and histology of the individual specimens assayed was also performed.

### *Preparation of cytosols*

All procedures were performed at 0–4°C. Approximately 200 mg of tissue was homogenised (Silverson) in 1:10 w/v of tissue buffer (pH 7.5) containing 20 mM Tris, 2 mM magnesium chloride, 1 mM calcium chloride, 10 mM calcium chloride, 16 mM HCl and 100 KIU Aprotinin $ml^{-1}$ (Bayer UK LTd). The homogenate was centrifuged at 105,000 *g* for 1 h (Sorval Superspeed 50) and the supernatant removed and used as cytosol.

### *Determination of protein content of cytosols*

A spectrophotometric method using Coomassie Brilliant Blue (Sigma) was employed (Bradford, 1976). Bovine serum albumen (Sigma) was used as a standard.

### *Cyclic AMP binding assay*

The cytosol (50 µl) was incubated in duplicate overnight at 4°C with 100 µl of 25 nM (to give a final concentration of 10 nM) 5′8-$^3$H-cAMP (Sp. Ac. 44.5–59 Ci $mmol^{-1}$ Amersham International) and 100 µl of assay buffer (55 mM potassium phosphate with the fresh addition of 11 mM theophylline) containing increasing final concentrations (0, 10, 20, 40, 80, 10,000 nM) or radio-inert cAMP (Sigma). Bound and free cAMP were then separated by filtration (Millipore HAWP 0.45 µm). Filters were washed in assay buffer containing 10 mM magnesium chloride and allowed to dry. Scintillant (5 ml, NE-260 Nuclear Enterprises) was added to each vial and incubated for 2 h at 37°C. The vials were then counted in

Correspondence: A.W. Bradbury.
Received 11 July 1990; and in revised form 25 September 1990.

**Table I** Colorectal cancers by sex of patient, site, Dukes' stage and grade

| *Site* | | | *Dukes' stage* | | | *Histological grade* | | |
|---|---|---|---|---|---|---|---|---|
| Site | Male | Female | Stage | Male | Female | Grade | Male | Female |
| CA | 3 | 7 | A | 2 | – | Moderate | 17 | 19 |
| AC | 2 | 1 | B | 15 | 17 | Poor | 6 | 8 |
| TC | – | 1 | C | 6 | 10 | | | |
| DC | 2 | 1 | | | | | | |
| SI | 5 | 13 | | | | | | |
| RE | 11 | 4 | | | | | | |

CA = caecum, AC = ascending colon, TC = transverse colon, DC = descending colon; SI = sigmoid, RE = rectum.

**Figure 1** Comparison of cAMP binding in 50 colorectal cancers and related adjacent and distant mucosa. O represent means and – the standard error. Means of all three samples statistically different by one-way ANOVA, $P<0.0001$.

**Figure 2** Correlation of cAMP binding in colorectal cancer and adjacent mucosa. 50 paired samples. Correlation coefficient $r = 0.64$, $P<0.0001$.

**Figure 3** Correlation of cAMP binding in colorectal cancer and distant mucosa. 50 paired samples. Correlation coefficient $r = 0.77$, $P<0.0001$.

a Tricarb liquid scintillation counter (Packard). Results were analysed by Scatchard analysis (Scatchard, 1949) and expressed as fmol cAMP bound per mg cytosol protein.

### Results

### *Cyclic AMP binding in tumour and mucosa*

Binding was detected in all specimens assayed. However, binding in the tumour (3366 ± s.e. 228, range 1012–8910) was significantly higher ($P<0.000001$ paired $t$-test) than binding in both adjacent mucosa (2298 ± s.e. 129, range 968–3546) and distant mucosa (2631 ± s.e. 146, range 1000–4807) ($P<0.00001$ paired $t$-test). By one-way ANOVA binding levels in all three specimens were significantly different from each other ($P<0.0001$). In addition, binding in the three specimens types were strongly inter-correlated. These data are presented in Figure 1, 2, 3 and 4.

### *Age and sex of patients*

There was no correlation between the age of the patient and the tumour level of cAMP (data not shown). Neither was binding in tumours from male patients (3563 ± s.e. 333) significantly different from that in tumours from female patients (3201 ± s.e. 318).

### *Site of tumour*

Cyclic AMP binding in both tumours and adjacent and distant mucosa was unrelated to the site of origin of that tumour within the colorectum (data not shown).

### *Stage of tumour*

Dukes' B tumours (3858 ± 294, range 1012–8910) had significantly higher binding ($P<0.005$ unpaired $t$-test) than Dukes' C tumours (2321 ± 205, range 1024–3871). The two Dukes' A tumours in the series had binding levels of 5619 and 2154. Adjacent and distant mucosa related to Dukes' B tumours also had significantly higher binding ($P<0.05$ and $P<0.001$ respectively) than the corresponding samples from mucosa related to Dukes' C tumours (Figure 5).

### *Grade of tumour*

With increasing de-differentiation there is a decrease in tumour cAMP binding although this does not attain statistical significance. Moderately differentiated tumours ($n = 36$, 3624 ± 314, range 1012–8910) had higher ($P = 0.07$) binding than those which were poorly differentiated ($n = 14$, 2708 ± 347, range 1415–4136) (Figure 6).

**Figure 4** Correlation of cAMP binding in colorectal cancer and adjacent mucosa. 50 paired samples. Correlation coefficient $r = 0.64$, $P < 0.0001$.

**Figure 5** Comparison of cAMP binding in colorectal cancers of different Dukes' stage. O represent means and – the standard error. Thirty-two Dukes' B tumours and 16 Dukes' C tumours. Binding in Dukes' B tumours was higher than binding in Dukes' C tumours ($P < 0.005$ by unpaired *t*-test). Both adjacent and distant mucosa from colons supporting the growth of Dukes' B tumours had higher binding than that from colons supporting the growth of Dukes' C tumours ($P < 0.05$ and $P < 0.0001$ by unpaired *t*-test respectively).

**Figure 6** Comparison of cAMP binding in tumours of different histological grade. O represent means and – the standard error. Thirty-six tumours graded as moderately differentiated have higher binding than 14 tumours graded as poorly differentiated ($P = 0.07$ by unpaired *t*-test).

### Discussion

This study is the first to show that human colorectal cancers have markedly elevated levels of cAMP binding proteins when compared to either adjacent or distant histologically benign mucosa. By contrast Alexandrov *et al.* (1986) reported low levels of cAMP and PK-A activity in villous adenomas and colorectal cancers when compared to normal mucosa. However, raised cAMP-BP levels may not reflect increased PK-A activity. Although the proportion of regulatory to catalytic sub-units is often been assumed to be equal, over-expression of abnormal cAMP binding proteins has been reported (Prashad, 1982).

This study also demonstrates that levels of cAMP binding in mucosa immediately adjacent to the tumours is lower than that in distant mucosa. Thus there appears to be a zone of depressed binding surrounding the tumour. It is possible that the tumour is releasing some, as yet unidentified, locally acting factor that is suppressing the surrounding mucosa. In this respect it has been shown that colorectal cancers are capable of secreting biologically active factors; for example Pommier (1988) has demonstrated the release of an autocrine growth factor by colorectal cancer cell lines. Alternatively, a 'transitional' mucosa surrounding colorectal cancers has been described characterised by inflammatory cell infiltrate, goblet and basal cell hyperplasia, mucosal ulceration and ischaemia (Lee, 1988). Such an abnormal mucosa may have different levels of cAMP binding *per se*. Cyclic AMP binding was higher in tumours of earlier stage and grade with levels falling as the tumour becomes more malignant. This is the first report of a relationship between cAMP binding protein expression and stage and grade of cancer. However, in breast cancer, although there is no correlation between cAMP binding protein levels and stage and grade of disease, high levels of binding proteins are of independent prognostic significance in predicting poor outcome in terms of disease-free interval and overall survival (Miller *et al.*, 1990). Despite stage and grade being general indicators of overall prognosis in colorectal cancer (Wiggers *et al.*, 1988), they are of limited use in predicting outcome for individual patients (Fielding *et al.*, 1986) reflecting the differing biology and behaviour of individual tumours. Likewise there is an almost 10-fold variation in cAMP binding between tumours in this series and a considerable overlap between Dukes' B (1012–8910) and Dukes' C (1024–3871) groups. It is intended to follow this group of patients to determine whether cAMP binding levels are related to overall prognosis.

Interestingly, cAMP binding in non-neoplastic mucosa from colons supporting Dukes' B tumours was significantly higher than that found in mucosa from colons with Dukes' C lesions. Thus stage dependent differences are present not only within malignant tissue, but also in benign mucosa adjacent to and distant from the cancer. These results indicate the possibility of pan-colonic changes in cAMP binding. Abnormalities in colonic mucosal proliferation have been frequently described in persons at increased risk of and in patients with colorectal cancer (Lipkin, 1988). Furthermore, increases in pan-colonic proliferative indices have been linked to the presence and size of colonic adenomas and cancers (Terpstra *et al.*, 1987). Lower levels PK-A activity have been reported in the proliferative compartment of colonic crypts (Schwartz *et al.*, 1988) and it may be that levels of cAMP binding found in this study reflect overall proliferative changes.

Because of the very significant differences in the level of expression of cAMP binding proteins between benign and malignant tissue in this study, it is relevant to discuss briefly the biology of cAMP binding proteins as it might relate to the malignant process. In addition to their role in regulating the kinase activity of PK-A, it has been postulated that cAMP binding proteins might have independent function in the regulation of cAMP gene transcription (Zwelling, 1988). For example, cAMP binding proteins have homology with DNA binding proteins and an initial report appeared to show that they possessed intrinsic Topoisomerase I activity (Constantinou *et al.*, 1985). However, more recently it has been possible to separate cAMP binding proteins from Topoisomerase activity (Shabb & Granner, 1988, Hunzicker-Dunn *et al.*, 1989). Despite these conflicting findings, cAMP binding proteins have been shown to enter the nucleus following PK-A activation (Tagliaferri *et al.*, 1988) and to associate with transcriptionally active chromatin during changes in gene expression (Sikorska *et al.*, 1988). Furthermore, cAMP has been shown to enhance the ability of cAMP binding proteins to bind oligonucleotide in a sequence-selective manner (Wu & Wang, 1989). Thus although many of the changes in cAMP dependent gene expression are due to the phosphorylation and activation of transcription factors by the catalytic subunit of PK-A (Ziff, 1990) there is increasing evidence that the regulatory cAMP binding subunits play an independent or modulating role. Interestingly, the ability of cAMP analogs to reverse the malignant phenotype of a number of cell lines including those derived from human colorectal cancer is associated with changes in the expression of cAMP binding proteins and the movement of binding proteins in to the nucleus (Cho-Chung *et al.*, 1989). Cyclic AMP binding proteins may therefore have an important role in controlling the expression of genes involved in development of the malignant state.

Irrespective of their mechanism of action, the results presented in this paper clearly demonstrate that an abnormality within the cAMP signalling system is associated with the development and possibly the progression of human colorectal cancers. This is the first report of such a finding and indicates that a defect in cellular control normally exerted through the cAMP system may be involved in colorectal carcinogenesis.

### References

ALEXANDROV, V.B., LEVITAN, M.Kh., MINAYEV, B.N., ESCHBA, I.R., VASILYEV, V.Y. & GRODZOVA, I.D. (1986). Measurement of cAMP levels and protein kinase activities as a diagnostic test for malignancy in the large bowel. *Vopr. Onkol.*, **32**, 45.

ALLY, S., TORTORA, G., CLAIR, T. & 7 others (1988). Selective modulation of protein kinase isoenzymes by the site-selective analog 8-chloroadenosine 3',5'-cyclic monophosphate provides a biological means for control of human colon cancer cell growth. *Proc. Natl Acad. Sci. USA*, **85**, 6319.

BRADFORD, M.M. (1976). A rapid and sensitive method for the quantitation of microgram quantities of protein utilising the principle of protein dye binding. *Anal. Biochem.*, **72**, 248.

CHO-CHUNG, Y.S., CLAIR, T.S., TAGLIAFERRI, P. & 7 others (1989). Site-selective cyclic AMP analogs as new biological tools in growth control, differentiation, and proto-oncogene activation. *Cancer Invest*, **7**, 161.

CONSTANTINOU, A.I., SQUINTO, S.P. & JUNGMANN, R.A. (1985). The phosphoform of the regulatory subunit RII of cAMP-dependent protein kinase possesses intrinsic topoisomerase activity. *Cell*, **42**, 429.

DERUBERTIS, F.R. & CRAVEN, P.A. (1980). Early alterations in rat colonic mucosal cyclic nucleotide metabolism and protein kinase activity induced by 1,2-dimethylhydrazine. *Cancer Res.*, **40**, 4589.

DUMONT, J.E., JAUNIAUX, J.-C. & ROGER, P.P. (1989). The cyclic AMP-mediated stimulation of cell proliferation. *Trends Biochem. Sci.*, **14**, 67.

FIELDING, L.P., PHILIPS, R.K.S., FRY, J.S. & HITTINGER, R. (1986). Prediction of outcome after curative resection for large bowel cancer. *Lancet*, **ii**, 904.

HUNZICKER-DUNN, M., MAIZELS, E.T., KERN, L.C., EKSTROM, R.C. & CONSTANTINOU, A.I. (1989). Separation of the complexes formed between the regulatory and catalytic subunits of cyclic adenosine monophosphate-dependent protein kinase and topoisomerase I activity in preovulatory follice-enriched immature rat ovaries. *Mol. Endocrinol.*, **3**, 780.

LEE, Y.-S. (1988). Background mucosal changes in colorectal carcinomas. *Cancer*, **61**, 1563.

LIPKIN, M. (1988). Biomarkers of increased susceptibility to gastrointestinal cancer: new application to studies of cancer prevention in human subjects. *Cancer Res.*, **48**, 235.

MILLER, W.R., ELTON, R.A., DIXON, J.M., CHETTY, U. & WATSON, D.M.A. (1990). Cyclic AMP binding proteins and prognosis in breast cancer. *Br. J. Cancer*, **61**, 263.

OLASHAW, N.E. & PLEDGER, W.J. (1988). Cellular mechanisms regulating proliferation. *Adv. Second Mess. Phosphoprotein Res.*, **22**, 139.

POMMIER, G., CULOUSCOU, J.M., GARROUSTE, F. & REMACLE-BONNET, M. (1988). CRGF: an autocrine growth factor associated with colorectal carcinomas. *Ann. NY Acad. Sci.*, **551**, 382.

PRASHAD, N. (1982). Induction of free cyclic-AMP-binding protein by dibutryl cyclic AMP in neuroblastoma cells. In *Protein Phosphorylation*, Rosen, O.M. & Krebbs, E.G. (eds) p. 159. Cold Spring Harbour.

ROESLER, W.J., VAN DEN BARK, G.R. & HANSON, R.W. (1988). Cyclic AMP and the induction of eukaryotic gene transcription. *J. Biol. Chem.*, **263**, 9063.

SCATCHARD, G. (1949). The attractions of proteins for small molecules and ions. *Ann. NY Acad. Sci.*, **51**, 660.

SCHWARTZ, B., FRASER, G.M., LEVY, J. & 4 others (1988). Differential distribution of protein kinases along the crypt-to-lumen regions of rat colonic epithelium. *Gut*, **29**, 1213.

SIKORSKA, M., WHITFIELD, J.F. & WALKER, P.R. (1988). The regulatory and catalytic subunits of cAMP-dependent protein kinases are associated with transcriptionally active chromatin during changes in gene expression. *J. Biol. Chem.*, **263**, 3005.

SHABB, J.B. & GRANNER, D.K. (1988). Separation of topoisomerase activity from the regulatory sub-unit of type II cyclic adenosine monophosphate-dependent protein kinase. *Mol. Endocrinol.*, **2**, 324.

SHENOLIKAR, S. (1988). Protein phosphorylation: hormones, drugs and bioregulation. *FASEB J.*, **2**, 2753.

TAGLIAFERRI, P., KATSAROS, D., CLAIR, T. & 10 others (1988). Synergistic inhibition of growth of breast and colon cancer cell lines by site-selective cyclic AMP analogs. *Cancer Res.*, **48**, 1642.

TAYLOR, S.S. (1989). cAMP-dependent protein kinase. *J. Biol. Chem.*, **264**, 8443.

TERPSTRA, O.T., VAN BLANKENSTEIN, M., DEES, J. & EILERS, G.A.M. (1987). Abnormal pattern of cell proliferation in the entire colonic mucosa of patients with colon adenoma or cancer. *Gastroenterology*, **92**, 704.

WIGGERS, T., ARENDS, J.W., SCHUTTE, B., VOLOVICS, L. & BOSMAN, F.T. (1988). A multivariate analysis of pathologic prognostic indicators in large bowel cancer. *Cancer*, **61**, 386.

WU, J.C. & WANG, J.H. (1989). Sequence-specific DNA binding to the regulatory sub-unit of cAMP-dependent protein kinase. *J. Biol. Chem.*, **264**, 9989.

ZIFF, E.B. (1990). Transcription factors: a new family gathers at the cAMP response site. *Trends in Genetics*, **6**, 69.

ZWELLING, L.A. (1988). Receptors and topoisomerase one protein, two functions, not always. *Mol. Endocrinol.*, **2**, 305.